# Impairment of cerebral vascular reactivity and resting blood flow in early-staged transgenic AD mice: *in vivo* optical imaging studies

**DOI:** 10.21203/rs.3.rs-3579916/v1

**Published:** 2023-11-11

**Authors:** Hyomin Jeong, Yingtian Pan, Firoz Akhter, Nora D. Volkow, Donghui Zhu, Congwu Du

**Affiliations:** 1 Department of Biomedical Engineering, Stony Brook University, Stony Brook, NY 11794, USA; 2 National Institute on Alcohol Abuse and Alcoholism, National Institutes of Health, Bethesda, MD 20857, USA

**Keywords:** Alzheimer’s disease, Neurodegenerative diseases, Neurovascular unit, Amyloid, Blood-Brain Barrier, Early diagnosis, Neurovascular dysfunction, OCT, Sex difference

## Abstract

**Background:**

Alzheimer’s disease (AD) is a neurodegenerative disorder with progressive cognitive decline in aging individuals that poses a significant challenge to patients due to an incomplete understanding of its etiology and lack of effective interventions. While “the Amyloid Cascade Hypothesis,” the abnormal accumulation of amyloid-β in the brain, has been the most prevalent theory for AD, mounting evidence from clinical and epidemiological studies suggest that defects in cerebral vessels and hypoperfusion appear prior to other pathological manifestations and might contribute to AD, leading to “the Vascular Hypothesis.” However, assessment of structural and functional integrity of the cerebral vasculature *in vivo* in the brain from AD rodent models has been challenging owing to the limited spatiotemporal resolution of conventional imaging technologies.

**Methods:**

We employed two *in vivo* imaging technologies, i.e., Dual-Wavelength Imaging (DWI) and Optical Coherence Tomography (OCT), to evaluate cerebrovascular reactivity (CVR; responsiveness of blood vessels to vasoconstriction as triggered by cocaine) in a relatively large field of view of the cortex *in vivo*, and 3D quantitative cerebrovascular blood flow (CBF) imaging in living transgenic AD mice at single vessel resolution.

**Results:**

Our results showed significantly impaired CVR and reduced CBF in basal state in transgenic AD mice compared to non-transgenic littermates in an early stage of AD progression. Changes in total hemoglobin (Δ[HbT]) in response to vasoconstriction were significantly attenuated in AD mice, especially in arteries and tissue, and the recovery time of Δ[HbT] after vasoconstriction was shorter for AD than WT in all types of vessels and cortical tissue, thereby indicating hypoperfusion and reduced vascular flexibility. Additionally, our 3D OCT images revealed that CBF velocities in arteries were slower and that the microvascular network was severely disrupted in the brain of AD mice.

**Conclusions:**

These results suggest significant vascular impairment in basal CBF and dynamic CVR in the neurovascular network in a rodent model of AD at an early stage of the disease. These cutting-edge *in vivo* optical imaging tools offer an innovative venue for detecting early neurovascular dysfunction in relation to AD pathology and pave the way for clinical translation of early diagnosis and elucidation of AD pathogenesis in the future.

## Background

Alzheimer’s disease (AD) results in gradual impairment of cognitive abilities and disruptive behavioral changes that contribute significantly to morbidity in aging populations. However, neuropathogenesis is not well understood and there are no effective treatments to prevent or treat AD. This knowledge gap reflects in part the heterogeneity of the disease and the diverse trajectories between patients including the severity of amyloid-β (Aβ) and cerebral amyloid angiopathy (CAA), as well as the difficulty in identifying the very early stage of AD. Many hypotheses have been postulated to explain the underlying cause of AD, the most popular one being “the Amyloid Cascade hypothesis,” which posits that abnormal accumulation of Aβ in the brain leads to neuronal dysfunction and eventually neurodegeneration based on numerous histologic and genetic data [1, 2]. However, the lack of a strong correlation between the severity of cognitive impairment and the level of Aβ deposition reported by some studies [3], has led to the hypothesis that senile Aβ plaques are the consequence of neurodegeneration, rather than the cause. A more recent hypothesis based on preclinical and clinical studies, “the Vascular hypothesis”, proposes that neurovascular dysfunction and vascular risk factors underlie AD [4–7]. Indeed, there is increasing evidence of vascular defects in the brain of patients in the early stages of AD [8–10] including cerebral hypoperfusion [11], leakage in the blood-brain barrier [12], and cerebral amyloid angiopathy that eventually leads to infarcts, microbleeds, and cerebral hemorrhage [13]. However, the majority of studies demonstrating structural alterations in the cerebrovasculature have used immunohistochemistry (IHC) in postmortem brain tissue from AD patients or transgenic AD animal models (summarized in Fisher et al. [14]) and could not capture dynamic changes nor three-dimensional (3D) spatial information of the vasculature. On the other hand, most *in vivo* studies assessing the effects of vascular risk factors such as chronic cerebral hypoperfusion and transient ischemia on brain changes in transgenic AD animals have only examined changes through inflammation and oxidative stress markers and behavioral tests (summarized in Scheffer et al. [7]), thereby providing limited insight into the actual vasculature changes in the brain of animal models of AD.

To emulate the heterogeneous pathological features that AD patients exhibit, several AD animal models have been developed with different AD transgenes inserted, such as amyloid-β-precursor protein (APP), presenilin (PSEN) 1, PSEN 2, apolipoprotein-E (APOE) 4, etc. leading to phenotypic variations [15, 16]. Recently, a human amyloid-β knock-in (hAb-KI) animal model was developed to express phenotypes that mimic the late-onset features in sporadic AD patients through a knock-in (KI) of a “humanized” Aβ sequence into the endogenous mouse APP protein [17]. Within this AD animal model, the changes in mRNA/protein levels, mitochondrial dynamics, an age-dependent decline in cognitive abilities, as well as changes in brain volume, etc. have been documented [17, 18] but vascular pathology has not been investigated yet.

Cerebrovascular reactivity (CVR) refers to the dynamic responsiveness of brain blood vessels to vasoconstrictive or vasodilatory stimuli, which can be used as a measure of the vascular physiological conditions within the brain [19]. Technologically, CVR can be examined through dynamic changes in cerebral blood flow (CBF) and/or cerebral blood volume (CBV) changes, which have been known as hemodynamic changes in vasculature. Various means of stimuli such as increased arterial CO_2_ partial pressure (i.e., hypercapnia) and exogenous chemicals have been used to assess pathologies of vascular-related diseases as well as AD [20–23]. Several clinical studies demonstrated that the progression of cognitive deficits in AD patients is aligned with the reduction of CVR and vascular contractibility [24–27]. However, this association is not yet conclusive as some studies reported that global CVR after hypercapnia was retained in AD [28, 29]. Also, studies to trigger vasoconstriction in AD patients are limited due to the risk of hypotension-induced ischemia [23]. Therefore, there is a need for further studies on CVR in AD patients and transgenic animals.

In clinical studies, mapping of CVR of the whole brain of AD patients is commonly achieved through functional magnetic resonance imaging (fMRI) for its advantages over positron emission tomography (PET) and single-photon emission computed tomography (SPECT) with non-radiation exposure and high repeatability [30]. Although the blood-oxygen-level-dependent (BOLD) [31] signal of fMRI can retrieve CBF signals with a high signal-to-noise ratio (SNR), it yields an indirect, relative CBF measurement that is potentially affected by other physiological parameters such as cerebral blood volume (CBV) and metabolic parameters such as cerebral metabolic rate of oxygen [32]. Also, it has been reported that BOLD signals are primarily from venous blood volume changes, not from arterial blood [33, 34], thereby providing an incomplete analysis of CVR across the neurovascular network. Alternatively, arterial spin labeling (ASL) [35] can be performed simultaneously with BOLD with the advantage of its direct, quantitative measurement of CBF; however, its SNR is limited [36]. While neural imaging tools such as PET, SPECT, and fMRI can provide insight into regional alterations of CVR in the whole brain of AD patients, their limited spatial resolution limits their ability to resolve individual cerebral vessels and the microvasculature, which are crucial to understanding cerebrovascular function associated with CVR abnormalities and AD progression [29].

To bridge this knowledge gap, we applied two cutting-edge *in vivo* imaging technologies, Dual-Wavelength Imaging (DWI) and Optical Coherent Tomography (OCT) to examine (i) CVR in response to a vasoconstrictive stimulus (i.e., acute cocaine challenge), (ii) cerebrovascular morphology including microvascular density, and (iii) basal CBF velocity (CBFv) of arteries, veins, and capillaries in the somatosensory cortex (SSC) of transgenic AD hA-beta-loxP-KI mice (hAb-KI) in comparison to non-transgenic littermates (WT). The research group that generated this AD mouse model reported that the hAb-KI mice exhibited an age-dependent increase in the level of insoluble Aβ, with the highest level at 18-22 months old, and displayed impaired performance in a cortical-dependent behavioral test from 14 months age [17]. These phenotypic alterations suggest that the hAb-KI mice can serve as a model for characterizing late-onset AD, and thus the middle age, 7-10 months old, was chosen to compare their differences with the age-matched WT before the emergence of cognitive deficits and severe accumulation of Aβ. The use of DWI developed in our lab [37] enabled real-time monitoring of dynamic vascular and tissue hemodynamic changes in response to cocaine challenge (1mg/kg, i.v., a vasoconstrictive stimulant [38]) with its high spatiotemporal resolution and large field of view (FOV). Additionally, three-dimensional (3D) mapping of the cerebrovasculature in the SSC was achieved at capillary resolution using our ultrahigh resolution OCT [39, 40], which simultaneously recorded the morphology of neurovascular trees by high-resolution optical coherence angiography (μOCA) along with the quantitative CBFv of these vessels by high-resolution optical Doppler tomography (μODT). Given that our OCT system can detect both arterial and venous volume changes with quantitative, not relative, CBFv, it allowed us to overcome the limitation of conventional imaging tools previously used to examine rodent models of AD.

Using these optical imaging techniques, we tested the following hypotheses: (1) CVR in AD mice would be impaired compared to WT mice *in vivo*, including an attenuation in vascular contractibility especially in arteries and (2) Basal CBFv would be lower in AD mice compared to WT with a lower microvascular density in the cortex of AD than control mice.

## Methods

### A. Animals

Transgenic hA-beta-loxP-KI mice (AD) with a humanized APP KI via AD-related three-point mutations (Jackson Laboratory, https://www.jax.org/strain/031050) and their non-transgenic littermates on a mixed C57BL/6J and C57BL/6NJ background (WT) were compared. Table 1 shows the number of animals used for each experimental protocol and drug challenge, imaging apparatus, and data processing that followed. All animals were housed with an ad libitum supply of food and water in a room where temperature, humidity, and light (12-hour light:dark cycle) were controlled. All the experimental procedures were approved by the Institutional Animal Care and Use committees of Stony Brook University and compliant with the National Institutes of Health Guide for the Care and Use of Laboratory Animals.

### B. The surgical procedure of cranial window implantation prior to *in vivo* imaging

All surgical apparatus and operation table were sterilized with 70% alcohol before surgery. The animal was anesthetized using a mixture of 1.5-2.5% isoflurane and oxygen, and its physiological changes including respiration rate and body temperature were monitored constantly during the surgical procedure to ensure proper anesthesia. The fur on the dorsal part of its head was shaved using a trimmer, and the animal was secured in a customized stereotaxic frame with a face mask for anesthesia. Lubricating eye drops were applied to protect its eyes, and 70% alcohol and povidone-iodine were applied topically to sterilize the head area. Its cortical skin was incised minimally along the sagittal suture and removed in a round shape to expose parietal bones and bregma. Then, the connective tissue on the skull was cleared by applying a hydrogen dioxide solution. The skull on the SSC was thinned in a rectangular shape (~2×2mm^2^) using a dental drill, and saline was constantly applied during the surgery to remove skull fragments. Once the skull was removed, a drop of saline was applied and a sterilized glass coverslip window (100μm thick) was fixed on top of the exposed brain surface with glue and dental cement [41].

### C. *In vivo* imaging of cerebrovascular reactivity via DWI and post-image processing

**Figure 1.**
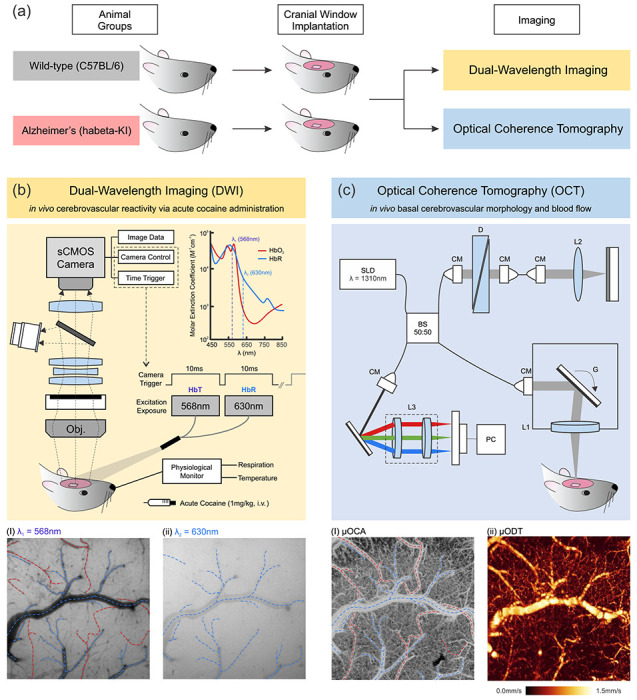
A schematic of research design. **(a)** Experimental Protocol: Transgenic AD mouse model (habeta-KI) and their non-transgenic littermates on a mixed C57BL/6J and C57BL/6NJ background were compared (left); a cranial window (~2 x 2 mm2) was implanted on the somatosensory cortex (SSC) of the mouse brain (middle); their cerebrovasculature was imaged using Dual-Wavelength Imaging (DWI) and Optical Coherence Tomography (OCT) separately (right). **(b)** Demonstration of DWI for imaging in vivo cerebrovascular reactivity via acute cocaine administration (1mg/kg, i.v.) as a physiological challenge; molar extinction coefficient spectrum demonstrates that changes in oxygenated hemoglobin (HbO2, red) and deoxygenated hemoglobin (HbR, blue) can be detected at λ1=568nm **(i)** and λ2=630nm **(ii)**, which can be used to quantify total hemoglobin level (HbT) in vasculatures as well in cortical tissue. **(c)** Illustration of OCT for imaging in vivo basal cerebrovascular morphology and blood flow imaging. **(i)** Ultra-high resolution optical coherence angiography (μOCA) shows cerebrovasculature with capillary resolution. Veins (blue dashed lines) and arteries (red dashed lines) were identified by comparing them to DWI images. **(ii)** Ultra-high resolution optical coherence Doppler tomography (μODT), simultaneously recorded with μOCA, provides quantitative cerebral blood flow velocity (CBFv). L1, 2, 3, achromatic lenses; CM, collimator; D, dispersion compensator; G, servo mirrors; BS, beam splitter; SLD, superluminescent diode.

*In vivo* imaging of hemodynamic changes, i.e., CVR, in the brain of WT and AD mice was carried out using the DWI developed in our laboratory [37, 39, 42]. The DWI consisted of a zoom fluorescence microscope (AZ100, Nikon) and a 2× Plan APO objective with sequential illumination of dual-wavelength LEDs (Spectra Light Engine, Lumencor) in a 10ms exposure for each light source was synchronized with an sCMOS camera (Zyla 5.5, Andor) to retrieve spectral images of the SSC at these two excitation wavelengths, λ_1_=568nm and λ_2_=630nm, simultaneously (Fig. 1b). Light exposure time and camera control were managed by a custom LabVIEW software with the high-speed digital triggers.

To compare the CVR of WT (n=8) and AD (n=12) mice *in vivo*, cocaine was selected as a pharmacological challenge for its known vasoconstricting effects [38, 39]. A catheter was placed into the tail vein and cocaine (1mg/kg) was acutely administered intravenously (i.v.) after 10 minutes of baseline imaging. After cocaine, the hemodynamic changes in the cortex were recorded continuously for 30 minutes for comparison.

Based on the molar extinction coefficient spectrum illustrated in Fig. 1b, the sequential image stacks collected at λ_1_=568nm (Fig. 1bi) are sensitive to show hemodynamic changes in both oxygenated hemoglobin (Δ[HbO_2_]) and deoxygenated hemoglobin (Δ[HbR]) in the cortex including in both veins and arteries, whereas the images obtained at λ_2_=630nm (Fig. 1bii) show a higher absorbance for [HbR] than that for [HbO_2_]. Accordingly, arteries and veins could be distinguished for quantification by comparing the two sets of image stacks that were simultaneously recorded via DWI, as demonstrated as red and blue traces in Fig. 1bi and bii, respectively.

Quantification of the dynamics of Δ[HbO_2_] and Δ[HbR] from the spectral images was achieved using the following formula [43], which is based on the assumption that Δ[HbO_2_] and Δ[HbO_2_] are the major sources for changes in light attenuation at the measured diffuse reflectance:

$$\left[\begin{array}{c} \Delta \left[HbO_{2}\left(t\right)\right]\\ \Delta \left[HbR\left(t\right)\right] \end{array}\right]={\left[\begin{array}{cc} \varepsilon_{HbO_{2}}^{\lambda_{1}} & \varepsilon_{HbR}^{\lambda_{1}}\\ \varepsilon_{HbO_{2}}^{\lambda_{2}} & \varepsilon_{HbR}^{\lambda_{2}} \end{array}\right]}^{-1}\times \left[\begin{array}{c} \frac{ln\left(R_{\lambda_{1}}\left(0\right)\right)/R_{\lambda_{1}}\left(t\right)}{L_{\lambda_{1}}\left(t\right)}\\ \frac{ln\left(R_{\lambda_{2}}\left(0\right)\right)/R_{\lambda_{2}}\left(t\right)}{L_{\lambda_{2}}\left(t\right)} \end{array}\right]$$

Here, $\varepsilon_{HbO_{2}}^{\lambda_{1}}$, $\varepsilon_{HbR}^{\lambda_{1}}$, $\varepsilon_{HbO_{2}}^{\lambda_{2}}$, and $\varepsilon_{HbR}^{\lambda_{2}}$ refer to the molar extinction coefficients of HbO_2_ and HbR at their respective wavelengths, λ_1_ and λ_2_. *R*_*λ*_1__ and *R*_*λ*_2__ indicate the measured diffuse reflectance matrices at time t, and *L*_*λ*_2__ are pathlengths of the light propagation. From the sum of Δ[HbO_2_] and Δ[HbR] acquired at the two wavelengths, changes in the total hemoglobin Δ[HbT], i.e. total blood volume in a given area of the cortex, can be quantified as a function of time. Three regions of interest (ROIs) each for arteries, veins, and tissue were manually selected from the spectral images, and their Δ[HbT] were quantified accordingly.

### D. *In vivo* imaging of basal cerebrovascular morphology and blood flow via OCT

A custom ultrahigh-resolution optical coherence tomography (μOCT) system developed in our laboratory [39, 40] consists of two functional extensions that enable simultaneous imaging of cerebrovascular morphology by ultra-high resolution optical coherence angiography (μOCA; Fig. 1ci) and a 3D cerebral vascular network with cerebral blood flow velocity (CBFv) quantification in the cortical brain by ultra-high resolution optical Doppler tomography (μODT; Fig. 1cii). As illustrated in Fig. 1c, an ultra-broadband light source (λ=1310nm and λ_FWHM_=220nm) illuminates a spectral-domain OCT engine with a 2×2 broadband fiber-optic Michelson interferometer, and it yields a relatively high axial resolution (~2.5μm) due to a short coherence length (*L*_*c*_ = 2*ln*(2)*λ*^2^/*π*Δ*λ*_*FWHM*_). In the sample arm of the system, a microscopic objective (*f*16mm/NA0.25) collimates the light over the cranial window transversely by a servo mirror, and a high-speed line scan InGaAs camera (2048-pixels, 145k-lines/s; GL2048, Sensors Unlimited) collects the transverse scans of backscattered light from the cortical vessels and tissue, resulting in the acquisition of 2D/3D μOCT in a transverse resolution of ~3μm. The camera was set to operate at 6k A-lines/sec for 3D μODT and 20k A-lines/sec for 3D μOCA.

CBFv was quantified by applying a custom Doppler flow reconstruction algorithm on μODT images [39, 40]. This reconstruction is based on the phase subtraction method (PSM) and phase intensity method (PIM). Simply, a phase shift induced by the Doppler flow of red blood cells in the vessels was detected between two consecutive A-scans, and it yielded the flow velocity (*ν*) of the red blood cells by the following equation [40, 44] :

$$v=\frac{\lambda_{0}\Delta \phi_{max}}{4\pi nTcos\theta }$$,

where λ_0_ refers to the central wavelength (0.00131mm), n is the refractive index (1.38), T is the time interval between two adjacent A-scans (0.000167sec), and ϴ is the angle between blood flow and light incidence (assumed ϴ=0). The DWI images acquired prior to OCT imaging were used to identify 1^st^ order arteries (the primary branch of arteries), and 2^nd^ order arteries, which are bifurcated from the 1^st^ order vessels in each animal. ROI selection was initially done based on the OCA images using ImageJ and then applied to the ODT images because vessels were not entirely visible in the ODT images if their flow was too low. Multiple ROIs were selected sequentially in a closely spaced arrangement along each artery to account for the variability of CBFv within each vessel and the measurements on the same artery were averaged. 7-10 per order of arteries in n=6 per group were analyzed and presented.

The diameters of 1^st^ and 2^nd^ order arteries were quantified using a customized MATLAB program that implemented the Frangi-Hessian filter [45]. An OCT image was first normalized and denoised of speckles by applying a median filter with a 3×1 window, and a “vesselness” score based on eigenvalues of the Hessian matrix was quantified at each pixel to generate a binarized segmentation [46]. Here, large and small vessels were segmented separately via the threshold segmentation method to avoid artifacts that commonly occur when the same Frangi-Hessian scale factor is applied to an image with a highly variable vessel profile [47]. The resulting vasculature masks of large and small vessels were combined, and values for diameter were assigned based on the Euclidean Distance Transformation algorithms [48]. Similar to the CBFv quantification, the 1^st^ and 2^nd^ order arteries were identified using the DWI images, and multiple ROIs were selected continuously along each artery from the processed OCA images. The multiple measurements on the same artery were averaged and the total mean of 6-9 arteries per order of arteries in n=6 per group is presented.

To compare the microvascular density in WT (n=8) and AD (n=6) mice, our lab developed a custom MATLAB program that processed an OCA image into a density map on a 0.00-0.15 scale. This program binarized and skeletonized the OCA image sequentially by employing the Frangi vesselness filter [49] and thinning-based skeletonization algorithm [50]. The resulting skeleton map *S* was further modified through morphological dilation and erosion operations to refine breakages in the skeletonized vessels caused by the inherent noise of OCA imaging. Then, the vascular fill factor (*FF*) at each pixel (x, y) (the measure of vascular density), was quantified over the whole image using a sliding window technique to generate a density map as shown in the following [51]:

$$FF=\frac{Total\;vessel\;pixel\;number}{Total\;pixel\;number\;of\;ROI}$$

$$FF_{\left(x,y\right)}=\frac{\left\|S_{\left(x,y,w\right)}\right\|}{w^{2}}$$

Here, *S*(x, y, w) refers to the image patch of size *w* = 60 at a given pixel on the skeleton map *S*. As a final step, a Gaussian filter (σ=3) was applied to the generated density map to achieve numerical stability and remove boundary artifacts. Then, three ROIs per post-processed image were manually selected in an area without large- or medium-sized vessels (>*ϕ* = 50μm), and their maximum and mean density were quantified. The resulting densities from the three ROIs per animal were averaged and the mean values across each group (n=9 for WT, n=6 for AD) were presented.

### E. Statistics

All data are presented as mean ± standard error of the mean (SEM). The differences between WT and AD mouse groups were analyzed using a two-tail Student’s t-test on SigmaStat software (Systat Software Inc.). To assess whether there was an interaction between sex and AD, two-way analysis of variance (ANOVA) with Bonferroni post-hoc tests was performed with “sex” and “groups (WT versus AD)” as sources of variation. The following criteria for p values were used: *p<0.05, **p<0.01, and ***p<0.001.

## Results

### 1. Impaired cerebrovascular reactivity (CVR) in response to cocaine in AD mice *in vivo*.

**Figure 2.**
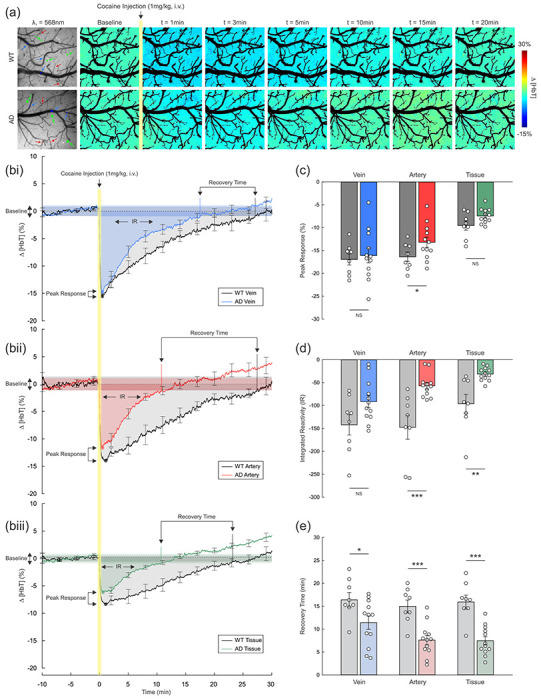
Comparison of cerebrovascular reactivity in response to cocaine between WT and AD mice *in vivo*. **(a)** Illustrative DWI images of dynamic changes of tissue total hemoglobin level (Δ[HbT]) in the SSC of WT and AD mice in response to an acute administration of cocaine (1mg/kg, i.v.) during imaging. The first column demonstrates original spectral images retrieved at λ1=568nm with three ROI selections each for veins (blue), arteries (red), and tissue (green). The images from the next seven columns show the ratio images of Δ[HbT] over the baseline (before cocaine) at different time points. The blood vessels were masked to demonstrate Δ[HbT] in tissue only. **(b)** Time course of mean Δ[HbT] (%) in response to cocaine in veins **(i)**, arteries **(ii)**, and tissue **(iii)** of WT (n=8) and AD mice (n=12). Peak response (%) refers to the maximum decrease in [HbT] after cocaine. IR, Integrated Reactivity, indicates the extent of cerebrovascular reactivity, calculated by integrating the curves until their respective recovery time. Recovery time shows how fast the vessels return to the range of their baseline HbT levels. Baseline and IR for AD mice were shaded in different colors (blue for veins, red for arteries, and green for tissue), and those for WT mice were shown in grey. **(c)** Comparison of the peak hemodynamic response of Δ[HbT] (%) to cocaine between WT (grey bars) and AD (colored bars) in cortical veins, arteries, and tissue, showing statistical significance in arteries between WT and AD animals (p=0.023). **(d)** IR of Δ[HbT] (a.u.) in WT (grey) and AD (colored), showing statistical significance in arteries (p<0.001) and tissue (p=0.002) within the cortexes. **(e)** Recovery time of Δ[HbT] (min) to the range of baseline in WT (grey) and AD (colored), showing statistical significance in all types of vessels, including veins and arteries (p=0.036 and p<0.001, respectively) as well in cortical tissue (p<0.001). NS, non-significance; *p<0.05; **p<0.01; ***p<0.001.

Figure 2 summarizes a comparative analysis of WT and AD mice in terms of CVR against the vasoconstrictive stimulus, cocaine (1mg/kg, i.v.). As a measure of CVR, changes in total hemoglobin (Δ[HbT]) that reflected the blood volume change, in vessels and cortical tissue were recorded for 10 minutes before and 30 minutes after cocaine administration. Figure 2a shows representative ratio images of Δ[HbT] in cortical tissue of WT versus AD mice over the baseline (before cocaine) at different time points (t=1, 3, 5, 10, 15, and 20 min after cocaine). It provides a visual demonstration of distinctive differences in cortical hemodynamic reactivities of the two groups. While the WT mouse showed a substantial decline in total blood volume (Δ[HbT]), as evidenced by the obvious change in color tone from green in baseline to blue within five minutes following cocaine injection, the AD mouse displayed temporary and less changes in [HbT]. Additionally, the color of the ratio images shows that the WT mouse returned to baseline around 15-20 minutes post-cocaine, whereas for the AD mouse, it returned within 5-10 minutes.

Figure 2b summarizes the mean time courses of quantitative Δ[HbT] (%) in veins (**i**), arteries (**ii**), and tissue (**iii**) separately over the 40 min period of imaging in WT (n=8) and AD (n=12) mice (ROIs = 3/veins, arteries, and tissue, respectively, per animal). For both groups, Δ[HbT] decreased sharply after cocaine injection and returned to baseline over time; however, the patterns of Δ[HbT] differed between the two groups. Three criteria, including i) peak response (Fig. 2c), ii) integrated reactivity (IR; Fig. 2d), and iii) recovery time (RT; Fig. 2e), were compared using the Δ[HbT] data to quantitatively evaluate CVR differences between the two groups. Specifically, the peak response was quantified by finding the maximum decrease in Δ[HbT] after cocaine administration. AD mice (−13.21±1.13%) exhibited a significantly lower peak response than WT mice (−16.37±1.04%) in arteries (p=0.023), but no differences for veins (−16.09±1.61% VS −16.97±1.22%, respectively for AD and WT groups; p=0.141) nor tissue (−7.45±0.47% VS −9.56±1.00%; p=0.074) (Fig. 2c). Additionally, IR, which reflects the extent or magnitude of the vascular reactivity, was calculated by integrating the time course of Δ[HbT] until its recovery. The results showed that IR of Δ[HbT] was significantly reduced in arteries (p<0.001), and cortical tissue (p=0.002), but not in veins (p=0/053) in AD compared to WT mice. IRs of AD mice were −91.92±13.40, −57.17±7.41, and −31.48±4.21 a.u., respectively for veins, arteries, and tissue, whereas for WT, they were −142.49±22.29, −147.97±26.09, −96.37±20.59 a.u.. The decrease in IR may signify structural changes and/or impaired functions in arteries and microvasculature of AD mice. The RT (min), which indicates the duration of time that Δ[HbT] took to return to its baseline, was accessed as a measure of the restorative ability of the cortical blood vessels. Interestingly, the RTs were shorter for AD mice than for WT mice, including 11.41±1.44min in veins (p=0.036), 7.61±1.03min in arteries (p<0.001), and 7.46±0.91min in tissue (p<0.001), compared to RTs for WT mice of 16.40±1.60min in veins, 14.96±1.38min in arteries, and 15.93±1.51min in tissue. Taken together, these results are indicative of impaired vascular reactivity in blood vessels of AD mice.

### 2. Female AD mice exhibited more severe impairment in vascular reactivity than males.

**Figure 3.**
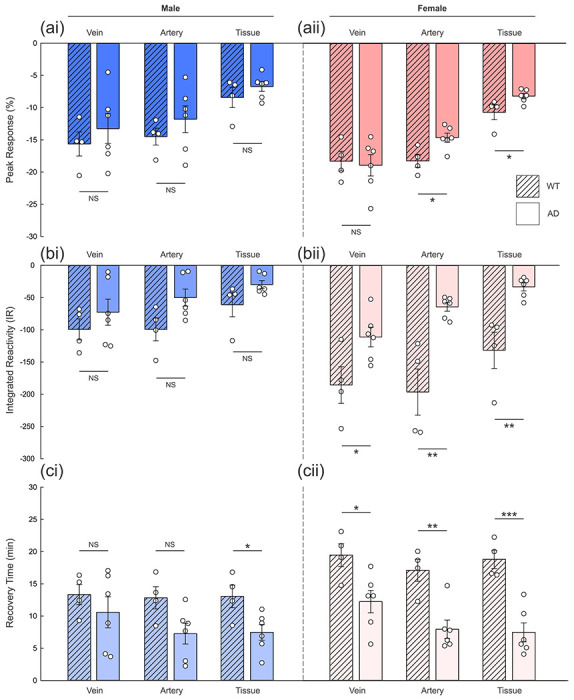
Sex-based differences in cerebrovascular reactivity *in vivo*. **(a)** Peak response of Δ[HbT] (%) in veins, arteries, and tissue of WT (n=4/sex; striped bars) and AD mice (n=6/sex; solid bars), showing no significant difference in males between WT and AD animals **(i)** but statistically significant difference in arteries between WT and AD females (p=0.017, in **ii).** **(b)** Integrated reactivity (IR) of Δ[HbT] (a.u.) in WT (n=4/sex; striped bars) and AD mice (n=6/sex; solid bars), showing statistical significance in all types of vessels and tissue in females (p=0.035, 0.002, and 0.003, respectively for veins, arteries, and tissue, in **ii)**. In male animals **(i)**, IRs of arteries in WT versus AD males did not show significant differences. **(c)** Recovery time of Δ[HbT] (min) to the range of baseline WT (n=4/sex; striped bars) and AD mice (n=6/sex; solid bars), showing statistical significance in all types of vessels and tissue in females (p=0.024, 0.003, and <0.001, respectively for veins, arteries, and tissue, in **ii)**. In male animals **(i)**, only tissue areas of AD returned significantly faster than those of WT (p=0.027) IRs of arteries in WT versus AD males did not show significant differences. NS, non-significance; *p<0.05; **p<0.01; ***p<0.001

While AD exhibits high prevalence in females [52], reports on its sex-biased differences in pathologies and pathogenesis have been equivocal, largely because there has been an insufficient number of sex-specific analyses in preclinical studies and sex has not been sufficiently recognized as a critical factor that may influence response to treatments in clinical trials of AD (summarized in Guo et al. [53]). Hence, we analyzed the CVR responses based on the sex difference of the animals. As shown in Fig. 3ai, males of AD and WT mice did not show any significant differences in peak response (%) in veins (−13.25±2.32% vs −15.64±1.87%, respectively for AD and WT; p=0.484), arteries (−11.79±2.07% vs −14.49±1.31%; p=0.360), and tissue (−6.72±−0.76% vs −8.41±0.31%; p=0.310). Similarly, the veins of female AD mice did not show a significant difference in the peak response compared to female WT (−18.93±1.66% vs −18.29±1.52%; p=0.796; Fig. 3aii); however, in arteries, female AD mice exhibited a lower peak response compared to female WT (−14.64±0.71% vs −18.24±1.01%; p=0.017). Also, the tissue of female AD mice had a significantly smaller response to cocaine injection as well (−8.18±0.42% vs −10.70±1.15%, respectively for AD and WT groups; p=0.043).

Furthermore, the female AD mice compared to female WT showed reduced IRs across the vascular trees including veins (−111.17±15.20 a.u. vs −185.67±28.48 a.u., for AD and WT; p=0.035), arteries (𢄡64.38±6.70a.u. vs −196.61±35.85 a.u.; p=0.002), and tissue (−33.29±6.32a.u. vs −131.72±28.16a.u.; p=0.003) (Fig. 3bii). In contrast, there were no significant differences in IRs between males with AD versus WT: −72.68±20.27 a.u. vs −99.31±16.26 a.u. for veins (p=0.376), −49.97±13.26 vs −99.33±17.71a.u. for arteries (p=0.052), and −29.67±6.06 vs −61.01±18.74a.u. for tissue (p=0.096) as shown in Fig. 3bi. The two-way ANOVA indicated significant sex by disease interactions for the arterial (F(1)=5.109, p=0.038) and tissue (F(1)=5.392, p=0.034) IRs, indicating greater severity on female than male mice.

In addition, the RTs of female AD (Fig. 3cii) were shorter in all types of vessels and tissue than those of female WT: 12.14α1.75min vs 19.46α1.77min for veins (p=0.024), 7.95α1.42min vs 17.10α1.77min for arteries (p=0.003), and 7.46α1.47min vs 18.80α1.41min for tissue (p<0.001), respectively for AD and WT. When males of WT and AD mice were compared, the RTs of cortical tissue areas of AD mice (7.47α1.23min) were shorter than those of WT mice (13.05α1.76min; p=0.027; Fig. 3ci) but not in veins (10.58α2.41min (AD) vs 13.33α1.58min (WT) on average; p=0.422) nor arteries (7.26α1.61 (AD) vs 12.83α1.73min (WT); p=0.051). Though the female AD mice appeared more impaired than male AD as compared with their wildtype counterpart the interaction between sex and AD was only significant for arterial and tissue IRs, which might reflect the small sample sizes.

### 3. AD decreased cerebrovascular blood flow velocities with no vessel diameter differences in arteries of AD with WT mice.

**Figure 4.**
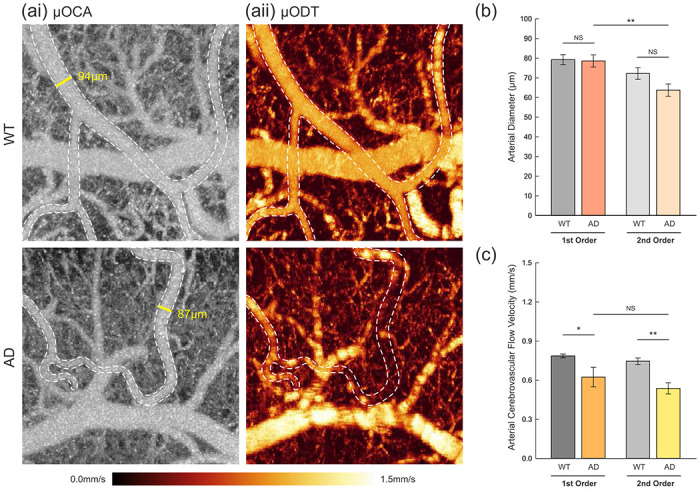
Vessel diameters and cerebrovascular blood flow velocity (CBFv) in arteries of WT and AD mice. **(a)** Representative angiograms μOCA **(i)** and simultaneously recorded quantitative CBFv (μODT in **ii)** of the SSC in the WT and AD brains. Despite the visible arteries in μOCA, a significant reduction of CBFv in the arteries of AD mice was commonly observed (white dashed lines). **(b)** Vessel diameter ϕ (μm, means ± SEM) of 1st and 2nd order arteries of WT and AD mice (n=6 per group; 6-9 arteries per order of arteries; multiple measurements on the same vessel were averaged). There were no statistically significant differences between the WT and AD mice for both 1st and 2nd arteries but the 2nd order arteries of AD were significantly narrower than those of 1st order (p=0.004). **(c)** Quantitative CBFv (mm/s, means ± SEM) of 1st and 2nd order arteries of WT and AD mice (n=6 per group; 7-10 arteries per order of arteries; multiple measurements on the same vessel were averaged). Statistically significant CBFv decreases were observed in both 1st and 2nd order arteries in the cortex of AD animals (p=0.038 and p=0.002, respectively). NS, non-significance; *p<0.05; **p<0.01.

To examine whether the blood flow in cortical arteries of AD mice is changed in the basal state, we took images of 3D vasculature of WT and AD mice (n=6 per group; 6-9 arteries per order of arteries via μOCA; Fig. 4ai) simultaneously with μODT to obtain quantitative CBFv measures (as demonstrated in Fig. 4aii). We observed that CBFv was decreased, and some arteries seemed occluded in AD mice (e.g., dashed trace of vessel visualized by μOCA (AD-ai), but for which no flow was detected shown in (AD-aii)) as compared to WT mice, despite having similar diameters, as illustrated in Fig. 4a. To quantitatively compare the arterial diameters of the two groups, the arteries identified using the DWI images were first categorized into 1^st^ order, primary branch, and 2^nd^ order, secondary branch bifurcated from the 1^st^ order for more comprehensive analysis. The vessel diameters (*ϕ;* μm) quantified from the μOCA images showed no significant differences (p = 0.868 and p = 0.085) between WT (79.28 ± 2.54 and 72.20 ± 2.94 μm) and AD mice (78.61 ± 3.09 and 63.77 ± 3.12 μm) for 1^st^ and 2^nd^ order arteries, respectively (Fig. 4b). Interestedly, the 2^nd^ order arteries of AD mice were significantly narrower than those of 1^st^ order arteries (p = 0.004), whereas there was no statistically significant difference for WT mice (p = 0.095).

We examined whether AD affected vascular blood flow by quantifying CBFv in the arteries for which diameters were quantified. As summarized in Fig. 4c, AD mice showed significant attenuation in CBFv compared to WT mice for both 1^st^ and 2^nd^ orders, suggesting a reduced basal circulation possibly due to partial occlusion of the vessels and/or narrowed vessels in high-order arteries. The mean CBFv for AD mice were 0.624 ± 0.075 and 0.537 ± 0.043 mm/s, and for WT, they were 0.787 ± 0.014 and 0.746 ± 0.025 mm/s for 1^st^ and 2^nd^ order arteries, respectively. Overall, simultaneous imaging with μOCA and μODT revealed that CBFv of arteries in the cortex were reduced significantly in AD mice, despite having negligible differences in arterial diameters compared to WT mice.

### 4. Microvascular density was decreased in AD mice.

**Figure 5.**
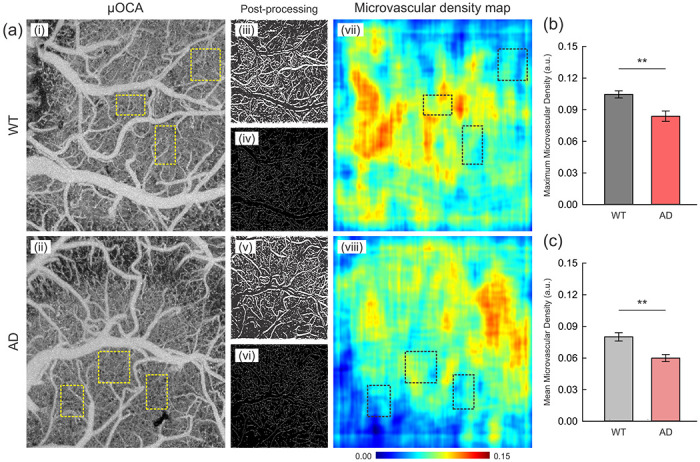
Evaluation of microvascular density between WT and AD mice. **(a)** Representative μOCA images of WT **(i)** and AD mice **(ii)** and microvascular density maps **(vii, viii)** converted from binarized **(iii, v)** and skeletonized **(iv, vi)** images via post-processing. Three microvascular areas devoid of large vessels were selected per animal (yellow dashed boxes, in **i, ii**), and maximum and mean densities in the selected ROIs (black dashed boxes) on the density maps **(vii, viii)** were extracted out of a 0.00-0.15 scale. **(b)** Comparison of the maximum microvascular density (a.u.) between WT (n=9) and AD mice (n=6), showing a statistically significant reduction in AD mice compared to WT (p=0.003). **(c)** Comparison of the mean microvascular density (a.u.) of WT and AD mice, showing a significant decrease of microvascular network in AD mice (p=0.005). **p<0.0

Neuronal activations are known to be coupled with increased CBF, especially in capillaries to meet the metabolic needs of cells, and thus impairment of capillary flow or density may lead to neuronal dysfunction [54, 55]. Hence, we assessed microvascular density in the cortex of WT (n=9, ROIs=3/animal) and AD mice (n=6, ROIs=3/animal) by post-processing the μOCA images into microvascular density maps (Fig. 5a). Maximum capillary densities in the selected areas without large- and medium-sized vessels (ϕ 50μm~) were significantly lower for AD (0.0839 ± 0.0049) than WT mice (0.1045 ± 0.0034, p=0.003) (Fig. 5b). Consistently, Figure 5c further demonstrates a decline in mean microvascular densities of AD (0.0600 ± 0.0054) compared to WT mice (0.0801 ± 0.0034, p=0.005), indicating a global disruption in the microvascular recruitment in the brain of AD compared to WT (Fig. 5c).

## Discussion

*In vivo* brain imaging of transgenic AD mice via the DWI and OCT strongly implicated that distinctive abnormalities in the cerebral vasculature emerge at an early stage of AD. Specifically, our data provided the following insights to deepen our understanding of vascular pathology in AD: (i) CVR was impaired in response to the vasoconstrictive stimulus, i.e., cocaine, in cortical arteries and tissue; (ii) CVR defects appeared stronger in females; (iii) arterial CBFv was significantly decreased in the resting state despite similar vessel diameters; and (iv) microvascular network density in the cortex was reduced. Taken together, these *in vivo* results are aligned with “the Vascular Hypothesis” and uniquely show vascular defects in the brain of AD mice in the early stage of AD progression.

Cerebral hypoperfusion and reduced CVR to vasodilators such as increased arterial CO_2_ partial pressure have been commonly used to evaluate patients with AD using MRI and PET [20, 21]; however, a close examination of individual vessels and their changes in blood volume *in vivo* has not been feasible due to the limited spatiotemporal resolution of these conventional imaging technologies. The DWI and OCT used in this study allowed us to overcome such shortcomings and identify morphological and functional alterations in the vasculature of a mouse AD model. Additionally, the effect of vasoconstrictors on the blood vessels of AD has been left largely underexplored. Given that the prevalence of stimulant drugs, which are vasoconstrictive [56, 57], is increasing, our data may also provide insights into the vascular effect from stimulant drug misuse that could increase the risk of AD [58].

The impaired CVR and decreased CBFv at the resting state observed in AD mice may be attributed to dysfunction of the neurovascular unit (NVU) prior to neurodegeneration. The NVU consists of cerebrovascular cells, including endothelial cells, smooth muscle cells, and pericytes, and non-vascular cells, including neurons and glia, and its proper functioning is crucial for the functional integrity of the brain as it orchestrates the supply of energy substrates and removal of metabolic byproducts in the brain. Especially, cerebral endothelial cells regulate CBF through endogenous vasodilators, such as nitric oxide (NO) and prostacyclin, as well as vasoconstrictors including endothelin and thromboxane [59]. Chemical signals emitted from endothelial cells, neurons, and astrocytes coordinate to constrict or dilate adjacent smooth muscle cells, which in turn counter changes in blood flow. Past studies have also reported mitochondrial abnormalities [60] and reduced expression of glucose transporter proteins (GLUT)-1 and - 3 in endothelial cells located in the cerebral cortex of AD [61], which preceded significant Aβ accumulation [62] and led to worsening of cognitive abilities [63]. These abnormalities in the endothelial cells may account for compromised NVU and consequent dysregulation of vascular flow as demonstrated in this study.

Vascular oxidative stress also leads to dysfunction of endothelial cells via the production of peroxynitrite from the interaction between reactive oxidative species (ROS) superoxide and nitric oxide (NO) [64]. Consequently, the lack of NO, the vasodilator from endothelial cells, from malfunctioning endothelial cells [65] contributes to vessel stiffness, which could induce shortened Δ[HbT] response to cocaine as observed above. Additionally, in an attempt to restore oxygen tension in the tissue, hypoxia-inducible transcription factors (HIFs) are activated. In the case of AD, the expression of HIFs [66] and the following upregulation of nicotinamide adenine dinucleotide phosphate oxidase enzyme NOX-2 [67] and ROS [68] have been shown to increase. This in turn results in oxidative cell damage and neuronal death [69], an event that is observed before the deposition of Aβ [70]. Interestingly, the increased expression of HIFs in neurons as a result of cerebral hypoperfusion and oxidative stress promotes the expression of β-secretase, an enzyme responsible for the abnormal cleavage of APP protein, and thus the production of Aβ, the pathological marker of AD [71, 72]. Hence, these cellular and molecular studies are also aligned with the suggested timeline of the symptom manifestation in AD development: early symptoms of vascular impairment and delayed emergence of Aβ plaques in the later stage of AD progression.

Aβ generated as a consequence of dysregulated NVU can trigger a vicious cycle of vascular and neuronal dysfunction along with further Aβ deposits because Aβ exerts vasoconstrictive effects through endothelial damage and induction of oxidative stress [73–75]. Aβ has been reported to selectively accumulate on the arterioles and cortical capillaries over venules [76–78], leading to the development of cerebral amyloid angiopathy (CAA) that can further weaken vasculature function [79]; this specificity on arteries and capillaries is consistent with the reduced CVR and CBFv especially in arteries and reduced microvascular density demonstrated in this study. Also, diameter constriction by Aβ has only been observed in capillaries, not arterioles or venules in transgenic AD mice *in vivo* via two-photon microscopy with intraluminal dyes [78]. Similarly, this study showed that there were no vascular diameter changes in the arteries of AD compared to WT animals, despite having a significant decrease in CBF in vascular networks. However, the underlying molecular mechanisms of decreased CBFv without changes in vessel diameters specifically in arteries remain unknown. Regardless, the vascular impairment imaged through our DWI and OCT holds important evidence on how vascular defects emerge at the early stage of AD and may undergo progressive decline as the NVU is further impaired and Aβ accumulates.

Furthermore, we have demonstrated that female AD mice exhibited more detectable CVR impairment than males when compared with age-matched WT animals, and sex-biased effect on AD was specifically observed in IRs of artery and cortical tissue. Likewise, female-specific changes during the progression of AD have been observed clinically, and a greater vulnerability of the female brains to AD development has been suggested by several studies (summarized by Guo et al. [80]). Female C57BL/6 mouse brains were shown to exhibit age-related alterations of gene expression earlier than males, and consequent early onset of hypometabolism and susceptibility to AD were reported [81]. Additionally, a single-nucleus transcriptomic analysis of AD patients revealed that a substantial number of female cells is represented in the cell subpopulations associated with AD, whereas male cells are mostly associated with non-pathological cell subpopulations. Further, gene activities of excitatory and inhibitory neurons appear to be downregulated in females [82], suggesting that transcriptional dysregulation in females may account for the sex-biased differences in CVR presented in this paper.

Most studies on changes in the vascular density of AD patients and transgenic AD mice demonstrated a decline in vascular density in alignment with disease progression (summarized in Fisher et al. [14]). The severe reduction in microvascular density in the cortex of AD mice observed in our study is consistent with these findings. However, OCT is unique in that it provides images of the vascular network in areas devoid of large- or medium-sized vessels (>*ϕ* = 50μm) within a large FOV. Also, prior methodology for quantifying vessel length density and vessel counts was largely limited to postmortem tissues and therefore highly dependent on staining protocols and confounding variables such as age variation from individual samples and possible tissue distortion in preparation. The feature of μOCA with label-free and high spatiotemporal resolution and automated segmentation technique presented in this study allowed us to detect the microvasculature in vivo and to quantitatively detect the decrease of microvascular density in the cortex of living hAb-KI mice in a large, 3D FOV without inherent limitations of contrast agents and fluorescent labeling.

Previously, a study by Bennett *et al*. demonstrated that there was no distinctive change in blood vessel volume in the cortex of 15-month-old APP/PS1 mice when assessed with *in vivo* two-photon imaging with fluorescein-dextran [83]. The discrepancy from our study could be attributed to different post-processing plugins to quantify density and the animal models used. We used the APP KI model (hA-beta-loxP-KI), which was recently developed to avoid phenotypic artifacts associated with APP overexpression [84] commonly observed in conventional transgenic mouse models such as the APP/PS1 model used by Bennet *et al*. The imperative difference in phenotypes between the two mouse models is that the dominant form of Aβ in the APP/PS1 model is Aβ42 [85], while that in hAb-KI is Aβ40, which has a vasoconstrictive effect in cerebral arterioles *in vitro* whereas Aβ42 does not *[*86*]*. Hence, this new AD mouse model with the KI approach may be more suitable for defining disease mechanisms concerning vascular defects [87].

One of the limitations of this study is that only one-time point (7-10 months old) was evaluated; therefore, longitudinal studies to examine the potential correlation between cognitive decline and CBF in AD mice and to pinpoint the age when the differences between AD and WT become distinctive will be helpful for understanding the progression of AD. Also in this study, we anesthetized mice with isoflurane to reduce motion artifacts, and future measurements in awake animals would reduce confounds from the vasodilatory effects of isoflurane [88]. Nevertheless, the data presented in this study using cutting-edge imaging techniques provides relevant information on the involvement of vascular alterations in AD.

## Conclusion

In summary, we show cerebrovascular dysfunction in early-stage AD mice using advanced optical imaging. The high spatial resolution allowed us to image the vascular network in a relatively large FOV and with high temporal resolution (at seconds). Our results add new evidence to “the Vascular Hypothesis” that cerebrovascular alterations observed at the early stage of AD progression may precede neurodegeneration in AD. However, further investigation of cellular/molecular mechanisms behind the alterations together with hemodynamic response and high-resolution angiography is needed to assess the biological causality between the health of the underlying NVU dysfunction observed in the AD mice.

## Figures and Tables

**Table 1.** Experimental Design for Animal Groups, Drug Challenge, Imaging, and Data Processing

Animal Groups	Drug Challenge	Imaging	Data Processing
**A** Wild-type (7-10 months; n = 8; 4 males and 4 females) **B** hA-beta-loxP-KI (7-10 months; n = 12; 6 males and 6 females)	Cocaine (1mg/kg, i.v.)	In vivo Dual-Wavelength Imaging (DWI; 6.5μm spatial resolution)	-Changes in total hemoglobin (Δ[HbT]) in response to drug challenge - Peak response, integrated reactivity, and return time of Δ[HbT]
**A** Wild-type (7-10 months; n = 9; 5 males and 4 females) **B** hA-beta-loxP-KI (7-10 months; n = 6; 3 males and 3 females)	N/A	In vivo Optical Coherence Tomography (OCT; <10 μm spatial resolution, penetration depth ~0.7mm)	- μODT (ultrahigh-resolution optical coherence Doppler tomography) for 3D imaging of cortical CBF networking and CBF velocity calculation - μOCA for 3D angiograph of blood vessels in the mouse brain and quantitative vessel diameters and densities

## Data Availability

The data sets generated and processed during the current study are available from the corresponding authors upon reasonable request.

## List of Abbreviations

AD: Alzheimer’s disease
Aβ: Amyloid-β
DWI: Dual-wavelength Imaging
OCT: Optical coherence tomography
μOCA: High-resolution optical coherence angiography
μODT: High-resolution optical Doppler tomography
CVR: Cerebrovascular reactivity
CBF: Cerebrovascular blood flow
CBFv: Cerebrovascular blood flow velocity
HbT: Total hemoglobin
HbO2: Oxygenated hemoglobin
HbR: Deoxygenated hemoglobin
CAA: Cerebral amyloid angiopathy
IHC: Immunohistochemistry
APP: Amyloid-β-precursor protein
PSEN: Presenilin
APOE: Apolipoprotein-E
KI: Knock-in
fMRI: Functional magnetic resonance imaging
PET: Positron emission tomography
SPECT: Single-photon emission computed tomography
BOLD: Blood-oxygen-level-dependent
SNR: Signal-to-noise ratio
CBV: Cerebral blood volume
ASL: Arterial spin labeling
SSC: Somatosensory cortex
WT: Wild type
FOV: Field of view
hAb-KI: hA-beta-loxP-KI mice
ROI: Region of interest
FWHM: Full width at half maximum
PSM: Phase subtraction method
PIM: Phase intensity method
FF: Fill factor
SEM: Standard error of the mean
ANOVA: Analysis of variance
IR: Integrated reactivity
RT: Return time
NVU: Neurovascular unit
GLUT: Glucose transporter proteins
ROS: Reactive oxidative species
HIF: Hypoxia-inducible transcription factor

## References

[1] Ballard C, Gauthier S, Corbett A, Brayne C, Aarsland D, Jones E: Alzheimer’s disease. The Lancet 2011, 377:1019–1031. 10.1016/S0140-6736(10)61349-9 21371747

[2] Hardy JA, Higgins GA: Alzheimer’s Disease: The Amyloid C ascade Hypothesis. Science 1992, 256:184–185. 1566067 10.1126/science.1566067

[3] Davis DG, Schmitt FA, Wekstein DR, Markesbery WR: Alzheimer Neuropathologic Alterations in Aged Cognitively Normal Subjects. Journal of Neuropathology & Experimental Neurology 1999, 58:376–388. 10218633 10.1097/00005072-199904000-00008

[4] de la Torre JC, Mussivand T: Can disturbed brain microcirculation cause Alzheimer’s disease? Neurol Res 1993, 15:146–153. 8103579 10.1080/01616412.1993.11740127

[5] Dickstein DL, Walsh J, Brautigam H, Stockton SD Jr., Gandy S, Hof PR: Role of vascular risk factors and vascular dysfunction in Alzheimer’s disease. Mt Sinai J Med 2010, 77:82–102. 20101718 10.1002/msj.20155 PMC2918901

[6] Rius-Pérez S, Tormos AM, Pérez S, Taléns-Visconti R: Vascular pathology: Cause or effect in Alzheimer disease? Neurología (English Edition) 2018, 33:112–120. 10.1016/j.nrl.2015.07.010 26385017

[7] Scheffer S, Hermkens DMA, Weerd Lvd, Vries HEd, Daemen MJAP: Vascular Hypothesis of Alzheimer Disease. Arteriosclerosis, Thrombosis, and Vascular Biology 2021, 41:1265–1283. 33626911 10.1161/ATVBAHA.120.311911

[8] Iturria-Medina Y, Sotero RC, Toussaint PJ, Mateos-Pérez JM, Evans AC, Weiner MW, Aisen P, Petersen R, Jack CR, Jagust W, Early role of vascular dysregulation on late-onset Alzheimer’s disease based on multifactorial data-driven analysis. Nature Communications 2016,7:11934. 10.1038/ncomms11934 PMC4919512 27327500

[9] Iturria-Medina Y, Carbonell FM, Sotero RC, Chouinard-Decorte F, Evans AC, Alzheimer’s Disease Neuroimaging I: Multifactorial causal model of brain (dis)organization and therapeutic intervention: Application to Alzheimer’s disease. Neuroimage 2017, 152:60–77. 28257929 10.1016/j.neuroimage.2017.02.058

[10] Jellinger KA: Alzheimer disease and cerebrovascular pathology: an update. Journal of Neural Transmission 2002, 109:813–836. 12111471 10.1007/s007020200068

[11] Wolters FJ, Zonneveld HI, Hofman A, van der Lugt A, Koudstaal PJ, Vernooij MW, Ikram MA, Heart-Brain Connection Collaborative Research G: Cerebral Perfusion and the Risk of Dementia: A Population-Based Study. Circulation 2017, 136:719–728. 28588075 10.1161/CIRCULATIONAHA.117.027448

[12] Wardlaw JM, Makin SJ, Valdés Hernández MC, Armitage PA, Heye AK, Chappell FM, Muñoz-Maniega S, Sakka E, Shuler K, Dennis MS, Thrippleton MJ: Blood-brain barrier failure as a core mechanism in cerebral small vessel disease and dementia: evidence from a cohort study. Alzheimer’s & Dementia 2016.

[13] Greenberg SM, Bacskai BJ, Hernandez-Guillamon M, Pruzin J, Sperling R, van Veluw SJ: Cerebral amyloid angiopathy and Alzheimer disease - one peptide, two pathways. Nat Rev Neurol 2020, 16:30–42. 31827267 10.1038/s41582-019-0281-2 PMC7268202

[14] Fisher RA, Miners JS, Love S: Pathological changes within the cerebral vasculature in Alzheimer’s disease: New perspectives. Brain Pathol 2022, 32:e13061. 35289012 10.1111/bpa.13061 PMC9616094

[15] Sanchez-Varo R, Mejias-Ortega M, Fernandez-Valenzuela JJ, Nuñez-Diaz C, Caceres-Palomo L, Vegas-Gomez L, Sanchez-Mejias E, Trujillo-Estrada L, Garcia-Leon JA, Moreno-Gonzalez I, Transgenic Mouse Models of Alzheimer&rsquo;s Disease: An Integrative Analysis. International Journal of Molecular Sciences 2022, 23:5404. 35628216 10.3390/ijms23105404 PMC9142061

[16] Jankowsky JL, Zheng H: Practical considerations for choosing a mouse model of Alzheimer’s disease. Molecular Neurodegeneration 2017, 12:89. 29273078 10.1186/s13024-017-0231-7 PMC5741956

[17] Baglietto-Vargas D, Forner S, Cai L, Martini AC, Trujillo-Estrada L, Swarup V, Nguyen MMT, Do Huynh K, Javonillo DI, Tran KM, Generation of a humanized Aβ expressing mouse demonstrating aspects of Alzheimer’s disease-like pathology. Nat Commun 2021, 12:2421. 33893290 10.1038/s41467-021-22624-z PMC8065162

[18] Kshirsagar S, Alvir RV, Hindle A, Kumar S, Vijayan M, Pradeepkiran JA, Reddy AP, Ramasubramanian B, Reddy PH: Early Cellular, Molecular, Morphological and Behavioral Changes in the Humanized Amyloid-Beta-Knock-In Mouse Model of Late-Onset Alzheimer’s Disease. Cells 2022, 11. 10.3390/cells11040733 PMC8869866 35203382

[19] Liu P, De Vis JB, Lu H: Cerebrovascular reactivity (CVR) MRI with CO2 challenge: A technical review. NeuroImage 2019, 187:104–115. 29574034 10.1016/j.neuroimage.2018.03.047 PMC6150860

[20] Chen JJ: Cerebrovascular-Reactivity Mapping Using MRI: Considerations for Alzheimer’s Disease. Frontiers in Aging Neuroscience 2018, 10. 10.3389/fnagi.2018.00170 PMC5996106 29922153

[21] Sleight E, Stringer MS, Marshall I, Wardlaw JM, Thrippleton MJ: Cerebrovascular Reactivity Measurement Using Magnetic Resonance Imaging: A Systematic Review. Frontiers in Physiology 2021, 12. 10.3389/fphys.2021.643468 PMC7947694 33716793

[22] Hayes G, Pinto J, Sparks SN, Wang C, Suri S, Bulte DP: Vascular smooth muscle cell dysfunction in neurodegeneration. Frontiers in Neuroscience 2022, 16. 10.3389/fnins.2022.1010164 PMC9684644 36440263

[23] Fierstra J, Sobczyk O, Battisti-Charbonney A, Mandell DM, Poublanc J, Crawley AP, Mikulis DJ, Duffin J, Fisher JA: Measuring cerebrovascular reactivity: what stimulus to use? J Physiol 2013, 591:5809–5821. 24081155 10.1113/jphysiol.2013.259150 PMC3872753

[24] Vicenzini E, Ricciardi MC, Altieri M, Puccinelli F, Bonaffini N, Di Piero V, Lenzi GL: Cerebrovascular reactivity in degenerative and vascular dementia: a transcranial Doppler study. Eur Neurol 2007, 58:84–89. 17565221 10.1159/000103642

[25] Cantin S, Villien M, Moreaud O, Tropres I, Keignart S, Chipon E, Le Bas JF, Warnking J, Krainik A: Impaired cerebral vasoreactivity to CO2 in Alzheimer’s disease using BOLD fMRI. Neuroimage 2011, 58:579–587. 21745581 10.1016/j.neuroimage.2011.06.070

[26] Richiardi J, Monsch AU, Haas T, Barkhof F, Van de Ville D, Radü EW, Kressig RW, Haller S: Altered cerebrovascular reactivity velocity in mild cognitive impairment and Alzheimer’s disease. Neurobiol Aging 2015, 36:33–41. 25146454 10.1016/j.neurobiolaging.2014.07.020

[27] Kim D, Hughes TM, Lipford ME, Craft S, Baker LD, Lockhart SN, Whitlow CT, Okonmah-Obazee SE, Hugenschmidt CE, Bobinski M, Jung Y: Relationship Between Cerebrovascular Reactivity and Cognition Among People With Risk of Cognitive Decline. Frontiers in Physiology 2021, 12. 10.3389/fphys.2021.645342 PMC8201407 34135768

[28] Rodell A, Aanerud J, Braendgaard H, Gjedde A: Low Residual CBF Variability in Alzheimer’s Disease after Correction for CO2 Effect. Frontiers in Neuroenergetics 2012, 4. 10.3389/fnene.2012.00008 PMC3389721 22783187

[29] Glodzik L, Randall C, Rusinek H, de Leon MJ: Cerebrovascular Reactivity to Carbon Dioxide in Alzheimer’s Disease. Journal of Alzheimer’s Disease 2013, 35:427–440. 10.3233/JAD-122011 PMC3776495 23478306

[30] Pinto J, Bright MG, Bulte DP, Figueiredo P: Cerebrovascular Reactivity Mapping Without Gas Challenges: A Methodological Guide. Frontiers in Physiology 2021, 11. 10.3389/fphys.2020.608475 PMC7848198 33536935

[31] Ogawa S, Menon RS, Tank DW, Kim SG, Merkle H, Ellermann JM, Ugurbil K: Functional brain mapping by blood oxygenation level-dependent contrast magnetic resonance imaging. A comparison of signal characteristics with a biophysical model. Biophysical Journal 1993, 64:803–812. 8386018 10.1016/S0006-3495(93)81441-3 PMC1262394

[32] Chen JJ, Pike GB: Global cerebral oxidative metabolism during hypercapnia and hypocapnia in humans: implications for BOLD fMRI. J Cereb Blood Flow Metab 2010, 30:1094–1099. 20372169 10.1038/jcbfm.2010.42 PMC2949195

[33] Lee S-P, Duong TQ, Yang G, Iadecola C, Kim S-G: Relative changes of cerebral arterial and venous blood volumes during increased cerebral blood flow: Implications for BOLD fMRI. Magnetic Resonance in Medicine 2001, 45:791–800. 11323805 10.1002/mrm.1107

[34] Lee S-P, Silva AC, Ugurbil K, Kim S-G: Diffusion-weighted spin-echo fMRI at 9.4 T: Microvascular/tissue contribution to BOLD signal changes. Magnetic Resonance in Medicine 1999, 42:919–928. 10542351 10.1002/(sici)1522-2594(199911)42:5<919::aid-mrm12>3.0.co;2-8

[35] Williams DS, Detre JA, Leigh JS, Koretsky AP: Magnetic resonance imaging of perfusion using spin inversion of arterial water. Proceedings of the National Academy of Sciences 1992, 89:212–216. 10.1073/pnas.89.1.212 PMC48206 1729691

[36] Bangen KJ, Restom K, Liu TT, Jak AJ, Wierenga CE, Salmon DP, Bondi MW: Differential age effects on cerebral blood flow and BOLD response to encoding: associations with cognition and stroke risk. Neurobiol Aging 2009, 30:1276–1287. 18160181 10.1016/j.neurobiolaging.2007.11.012 PMC2804245

[37] Yuan Z, Luo Z, Volkow ND, Pan Y, Du C: Imaging separation of neuronal from vascular effects of cocaine on rat cortical brain in vivo. NeuroImage 2011, 54:1130–1139. 20804849 10.1016/j.neuroimage.2010.08.045 PMC2997146

[38] Rapoport RM, Yoon S, Zuccarello M: Cocaine Constrictor Mechanisms of the Cerebral Vasculature. Journal of Cardiovascular Pharmacology 2016, 67. 10.1097/FJC.0000000000000361 26771152

[39] Ren H, Du C, Yuan Z, Park K, Volkow ND, Pan Y: Cocaine-induced cortical microischemia in the rodent brain: clinical implications. Mol Psychiatry 2012, 17:1017–1025. 22124273 10.1038/mp.2011.160 PMC3934297

[40] You J, Du C, Volkow ND, Pan Y: Optical coherence Doppler tomography for quantitative cerebral blood flow imaging. Biomed Opt Express 2014, 5:3217–3230. 25401033 10.1364/BOE.5.003217 PMC4230874

[41] Park K, You J, Du C, Pan Y: Cranial window implantation on mouse cortex to study microvascular change induced by cocaine. Quant Imaging Med Surg 2015, 5:97–107. 25694959 10.3978/j.issn.2223-4292.2014.11.31 PMC4312290

[42] Chen W, Park K, Volkow ND, Pan Y, Du C: Cocaine-Induced Abnormal Cerebral Hemodynamic Responses to Forepaw Stimulation Assessed by Integrated Multi-Wavelength Spectroimaging and Laser Speckle Contrast Imaging. IEEE Journal of Selected Topics in Quantum Electronics 2016, 22:146–153. 10.1109/JSTQE.2015.2503319 PMC4991560 27551166

[43] Luo Z, Yuan Z, Pan Y, Du C: Simultaneous imaging of cortical hemodynamics and blood oxygenation change during cerebral ischemia using dual-wavelength laser speckle contrast imaging. Opt Lett 2009, 34:1480–1482. 19412312 10.1364/ol.34.001480

[44] Zhao Y, Chen Z, Saxer C, Xiang S, de Boer JF, Nelson JS: Phase-resolved optical coherence tomography and optical Doppler tomography for imaging blood flow in human skin with fast scanning speed and high velocity sensitivity. Opt Lett 2000, 25:114–116. 18059800 10.1364/ol.25.000114

[45] Yousefi S, Liu T, Wang RK: Segmentation and quantification of blood vessels for OCT-based micro-angiograms using hybrid shape/intensity compounding. Microvasc Res 2015, 97:37–46. 25283347 10.1016/j.mvr.2014.09.007 PMC4275398

[46] Li A, You J, Du C, Pan Y: Automated segmentation and quantification of OCT angiography for tracking angiogenesis progression. Biomed Opt Express 2017, 8:5604–5616. 29296491 10.1364/BOE.8.005604 PMC5745106

[47] You J, Pan C, Park K, Li A, Du C: In vivo detection of tumor boundary using ultrahigh-resolution optical coherence angiography and fluorescence imaging. Journal of Biophotonics 2020, 13:e201960091. 31778294 10.1002/jbio.201960091 PMC7446292

[48] Shih FYC, Mitchell OR: A mathematical morphology approach to Euclidean distance transformation. IEEE Transactions on Image Processing 1992, 1:197–204. 18296154 10.1109/83.136596

[49] Frangi AF, Niessen WJ, Vincken KL, Viergever MA: Multiscale vessel enhancement filtering. In Medical Image Computing and Computer-Assisted Intervention—MICCAI’98: First International Conference Cambridge, MA, USA, October 11–13, 1998 Proceedings 1. Springer; 1998:130–137.

[50] Lam L, Lee S-W, Suen CY: Thinning methodologies-a comprehensive survey. IEEE Transactions on Pattern Analysis & Machine Intelligence 1992, 14:869–885.

[51] Du C, Volkow ND, You J, Park K, Allen CP, Koob GF, Pan Y: Cocaine-induced ischemia in prefrontal cortex is associated with escalation of cocaine intake in rodents. Mol Psychiatry 2020, 25:1759–1776. 30283033 10.1038/s41380-018-0261-8 PMC6447479

[52] 2022 Alzheimer’s disease facts and figures. Alzheimer’s & Dementia 2022, 18:700–789. 10.1002/alz.12638 35289055

[53] Guo L, Zhong MB, Zhang L, Zhang B, Cai D: Sex Differences in Alzheimer’s Disease: Insights From the Multiomics Landscape. Biological Psychiatry 2022, 91:61–71. 33896621 10.1016/j.biopsych.2021.02.968 PMC8996342

[54] Itoh Y, Suzuki N: Control of brain capillary blood flow. J Cereb Blood Flow Metab 2012, 32:1167–1176. 22293984 10.1038/jcbfm.2012.5 PMC3390803

[55] LaManna JC, Chavez JC, Pichiule P: Structural and functional adaptation to hypoxia in the rat brain. Journal of Experimental Biology 2004, 207:3163–3169. 15299038 10.1242/jeb.00976

[56] Broadley KJ: The vascular effects of trace amines and amphetamines. Pharmacol Ther 2010, 125:363–375. 19948186 10.1016/j.pharmthera.2009.11.005

[57] Bachi K, Mani V, Jeyachandran D, Fayad ZA, Goldstein RZ, Alia-Klein N: Vascular disease in cocaine addiction. Atherosclerosis 2017, 262:154–162. 28363516 10.1016/j.atherosclerosis.2017.03.019 PMC5757372

[58] Tzeng NS, Chien WC, Chung CH, Chang HA, Kao YC, Liu YP: Association between amphetamine-related disorders and dementia-a nationwide cohort study in Taiwan. Ann Clin Transl Neurol 2020, 7:1284–1295. 32608133 10.1002/acn3.51113 PMC7448166

[59] Faraci FM, Heistad DD: Regulation of the cerebral circulation: role of endothelium and potassium channels. Physiol Rev 1998, 78:53–97. 9457169 10.1152/physrev.1998.78.1.53

[60] Baloyannis SJ, Baloyannis IS: The vascular factor in Alzheimer’s disease: a study in Golgi technique and electron microscopy. J Neurol Sci 2012, 322:117–121. 22857991 10.1016/j.jns.2012.07.010

[61] Kalaria RN, Harik SI: Reduced glucose transporter at the blood-brain barrier and in cerebral cortex in Alzheimer disease. J Neurochem 1989, 53:1083–1088. 2769254 10.1111/j.1471-4159.1989.tb07399.x

[62] Merlini M, Meyer EP, Ulmann-Schuler A, Nitsch RM: Vascular β-amyloid and early astrocyte alterations impair cerebrovascular function and cerebral metabolism in transgenic arcAβ mice. Acta Neuropathol 2011, 122:293–311. 21688176 10.1007/s00401-011-0834-y PMC3168476

[63] Winkler EA, Nishida Y, Sagare AP, Rege SV, Bell RD, Perlmutter D, Sengillo JD, Hillman S, Kong P, Nelson AR, GLUT1 reductions exacerbate Alzheimer’s disease vasculo-neuronal dysfunction and degeneration. Nat Neurosci 2015, 18:521–530. 25730668 10.1038/nn.3966 PMC4734893

[64] Villa LM, Salas E, Darley-Usmar VM, Radomski MW, Moncada S: Peroxynitrite induces both vasodilatation and impaired vascular relaxation in the isolated perfused rat heart. Proc Natl Acad Sci U S A 1994, 91:12383–12387. 7809045 10.1073/pnas.91.26.12383 PMC45442

[65] Kelleher RJ, Soiza RL: Evidence of endothelial dysfunction in the development of Alzheimer’s disease: Is Alzheimer’s a vascular disorder? Am J Cardiovasc Dis 2013, 3:197–226. 24224133 PMC3819581

[66] Grammas P, Tripathy D, Sanchez A, Yin X, Luo J: Brain microvasculature and hypoxia-related proteins in Alzheimer’s disease. Int J Clin Exp Pathol 2011, 4:616–627. 21904637 PMC3160613

[67] Shimohama S, Tanino H, Kawakami N, Okamura N, Kodama H, Yamaguchi T, Hayakawa T, Nunomura A, Chiba S, Perry G, Activation of NADPH Oxidase in Alzheimer’s Disease Brains. Biochemical and Biophysical Research Communications 2000, 273:5–9. 10873554 10.1006/bbrc.2000.2897

[68] Kishida KT, Pao M, Holland SM, Klann E: NADPH oxidase is required for NMDA receptor-dependent activation of ERK in hippocampal area CA1. J Neurochem 2005, 94:299–306. 15998281 10.1111/j.1471-4159.2005.03189.x PMC3544193

[69] Sorce S, Krause K-H: NOX Enzymes in the Central Nervous System: From Signaling to Disease. Antioxidants & Redox Signaling 2009, 11:2481–2504. 19309263 10.1089/ars.2009.2578

[70] Nunomura A, Perry G, Aliev G, Hirai K, Takeda A, Balraj EK, Jones PK, Ghanbari H, Wataya T, Shimohama S, Oxidative Damage Is the Earliest Event in Alzheimer Disease. Journal of Neuropathology & Experimental Neurology 2001, 60:759–767. 11487050 10.1093/jnen/60.8.759

[71] Zhang X, Zhou K, Wang R, Cui J, Lipton SA, Liao FF, Xu H, Zhang YW: Hypoxia-inducible factor 1alpha (HIF-1alpha)-mediated hypoxia increases BACE1 expression and beta-amyloid generation. J Biol Chem 2007, 282:10873–10880. 17303576 10.1074/jbc.M608856200

[72] Kitaguchi H, Tomimoto H, Ihara M, Shibata M, Uemura K, Kalaria RN, Kihara T, Asada-Utsugi M, Kinoshita A, Takahashi R: Chronic cerebral hypoperfusion accelerates amyloid beta deposition in APPSwInd transgenic mice. Brain Res 2009, 1294:202–210. 19646974 10.1016/j.brainres.2009.07.078

[73] Deane R, Du Yan S, Submamaryan RK, LaRue B, Jovanovic S, Hogg E, Welch D, Manness L, Lin C, Yu J, RAGE mediates amyloid-beta peptide transport across the blood-brain barrier and accumulation in brain. Nat Med 2003, 9:907–913. 12808450 10.1038/nm890

[74] Thomas T, Thomas G, McLendon C, Sutton T, Mullan M: beta-Amyloid-mediated vasoactivity and vascular endothelial damage. Nature 1996, 380:168–171. 8600393 10.1038/380168a0

[75] Iadecola C, Zhang F, Niwa K, Eckman C, Turner SK, Fischer E, Younkin S, Borchelt DR, Hsiao KK, Carlson GA: SOD1 rescues cerebral endothelial dysfunction in mice overexpressing amyloid precursor protein. Nat Neurosci 1999, 2:157–161. 10195200 10.1038/5715

[76] Dorr A, Sahota B, Chinta LV, Brown ME, Lai AY, Ma K, Hawkes CA, McLaurin J, Stefanovic B: Amyloid-β-dependent compromise of microvascular structure and function in a model of Alzheimer’s disease. Brain 2012, 135:3039–3050. 23065792 10.1093/brain/aws243

[77] Weller RO, Nicoll JA: Cerebral amyloid angiopathy: pathogenesis and effects on the ageing and Alzheimer brain. Neurol Res 2003, 25:611–616. 14503015 10.1179/016164103101202057

[78] Nortley R, Korte N, Izquierdo P, Hirunpattarasilp C, Mishra A, Jaunmuktane Z, Kyrargyri V, Pfeiffer T, Khennouf L, Madry C, Amyloid β oligomers constrict human capillaries in Alzheimer’s disease via signaling to pericytes. Science 2019, 365. 10.1126/science.aav9518. PMC6658218 31221773

[79] Serrano-Pozo A, Frosch MP, Masliah E, Hyman BT: Neuropathological alterations in Alzheimer disease. Cold Spring Harb Perspect Med 2011, 1:a006189. 22229116 10.1101/cshperspect.a006189 PMC3234452

[80] Guo L, Zhong MB, Zhang L, Zhang B, Cai D: Sex Differences in Alzheimer’s Disease: Insights From the Multiomics Landscape. Biol Psychiatry 2022, 91:61–71. 33896621 10.1016/j.biopsych.2021.02.968 PMC8996342

[81] Zhao L, Mao Z, Woody SK, Brinton RD: Sex differences in metabolic aging of the brain: insights into female susceptibility to Alzheimer’s disease. Neurobiol Aging 2016, 42:69–79. 27143423 10.1016/j.neurobiolaging.2016.02.011 PMC5644989

[82] Mathys H, Davila-Velderrain J, Peng Z, Gao F, Mohammadi S, Young JZ, Menon M, He L, Abdurrob F, Jiang X, Single-cell transcriptomic analysis of Alzheimer’s disease. Nature 2019, 570:332–337. 31042697 10.1038/s41586-019-1195-2 PMC6865822

[83] Bennett RE, Robbins AB, Hu M, Cao X, Betensky RA, Clark T, Das S, Hyman BT: Tau induces blood vessel abnormalities and angiogenesis-related gene expression in P301L transgenic mice and human Alzheimer’s disease. Proc Natl Acad Sci U S A 2018, 115:E1289–e1298. 29358399 10.1073/pnas.1710329115 PMC5819390

[84] Sasaguri H, Nilsson P, Hashimoto S, Nagata K, Saito T, De Strooper B, Hardy J, Vassar R, Winblad B, Saido TC: APP mouse models for Alzheimer’s disease preclinical studies. The EMBO Journal 2017, 36:2473–2487. 28768718 10.15252/embj.201797397 PMC5579350

[85] Maia LF, Kaeser SA, Reichwald J, Hruscha M, Martus P, Staufenbiel M, Jucker M: Changes in Amyloid-β; and Tau in the Cerebrospinal Fluid of Transgenic Mice Overexpressing Amyloid Precursor Protein. Science Translational Medicine 2013, 5:194re192–194re192. 10.1126/scitranslmed.3006446 23863834

[86] Niwa K, Carlson GA, Iadecola C: Exogenous A beta1-40 reproduces cerebrovascular alterations resulting from amyloid precursor protein overexpression in mice. J Cereb Blood Flow Metab 2000, 20:1659–1668. 11129782 10.1097/00004647-200012000-00005

[87] Xia D, Lianoglou S, Sandmann T, Calvert M, Suh JH, Thomsen E, Dugas J, Pizzo ME, DeVos SL, Earr TK, Novel App knock-in mouse model shows key features of amyloid pathology and reveals profound metabolic dysregulation of microglia. Molecular Neurodegeneration 2022, 17:41. 35690868 10.1186/s13024-022-00547-7 PMC9188195

[88] Park K, Chen W, Volkow ND, Allen CP, Pan Y, Du C: Hemodynamic and neuronal responses to cocaine differ in awake versus anesthetized animals: Optical brain imaging study. NeuroImage 2019, 188:188–197. 30513396 10.1016/j.neuroimage.2018.11.062 PMC6435283

